# Supplementary material for: Closed-loop recycling of tough epoxy supramolecular thermosets constructed with hyperbranched topological structure
Source: Nat Commun. 2024 Jun 7;15:4869. doi: 10.1038/s41467-024-49272-3 (PMC11161517; doi:10.1038/s41467-024-49272-3)
Supplement: Supplementary file 1 — Supplementary Information [file 41467_2024_49272_MOESM1_ESM.pdf]

Supplementary Information

Closed-Loop Recycling of Tough Epoxy Supramolecular Thermosets Constructed with Hyperbranched Topological Structure

Junheng Zhang[a*,b], Can Jiang[a], Guoyan Deng[a], Mi Luo[c], Bangjiao Ye[c], Hongjun Zhang[c*], Menghe Miao[d], Tingcheng Li[a], Daohong Zhang[a*]

[a]Hubei R&D Center of Hyperbranched Polymers Synthesis and Applications, South-Central Minzu University, Wuhan 430074, China.

[b]Guangdong Provincial Laboratory of Chemistry and Fine Chemical Engineering Jieyang Center, Jieyang 515200, China

[c]State Key Laboratory of Particle Detection and Electronics, University of Science and Technology of China, Hefei 230026, China.

[d]Department of Mechanical Engineering, The University of Melbourne, Grattan Street, Parkville, Victoria 3010, Australia.

*E-mail: mcjhzhang@gmail.com, hjzhang8@ustc.edu.cn, daohong.zhang@scuec.edu.cn

1. SUPPORTING FIGURES AND TABLES

1. SUPPORTING FIGURES AND TABLES

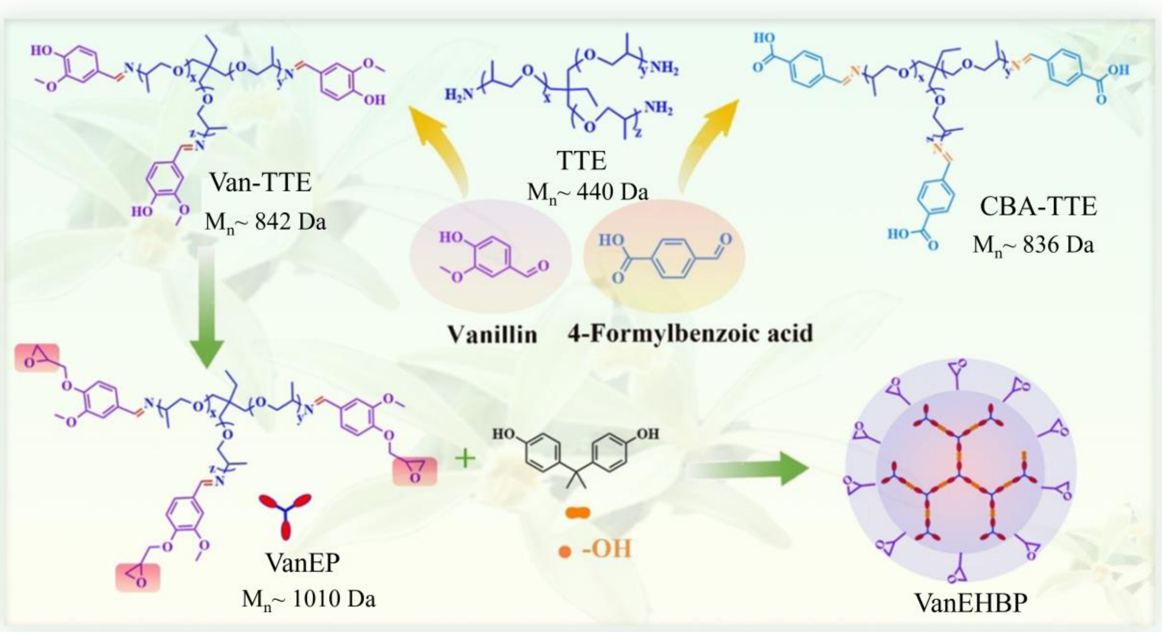

Supplementary Figure 1. Synthetic approaches for VanEP, VanEHBP and CBA-TTE.

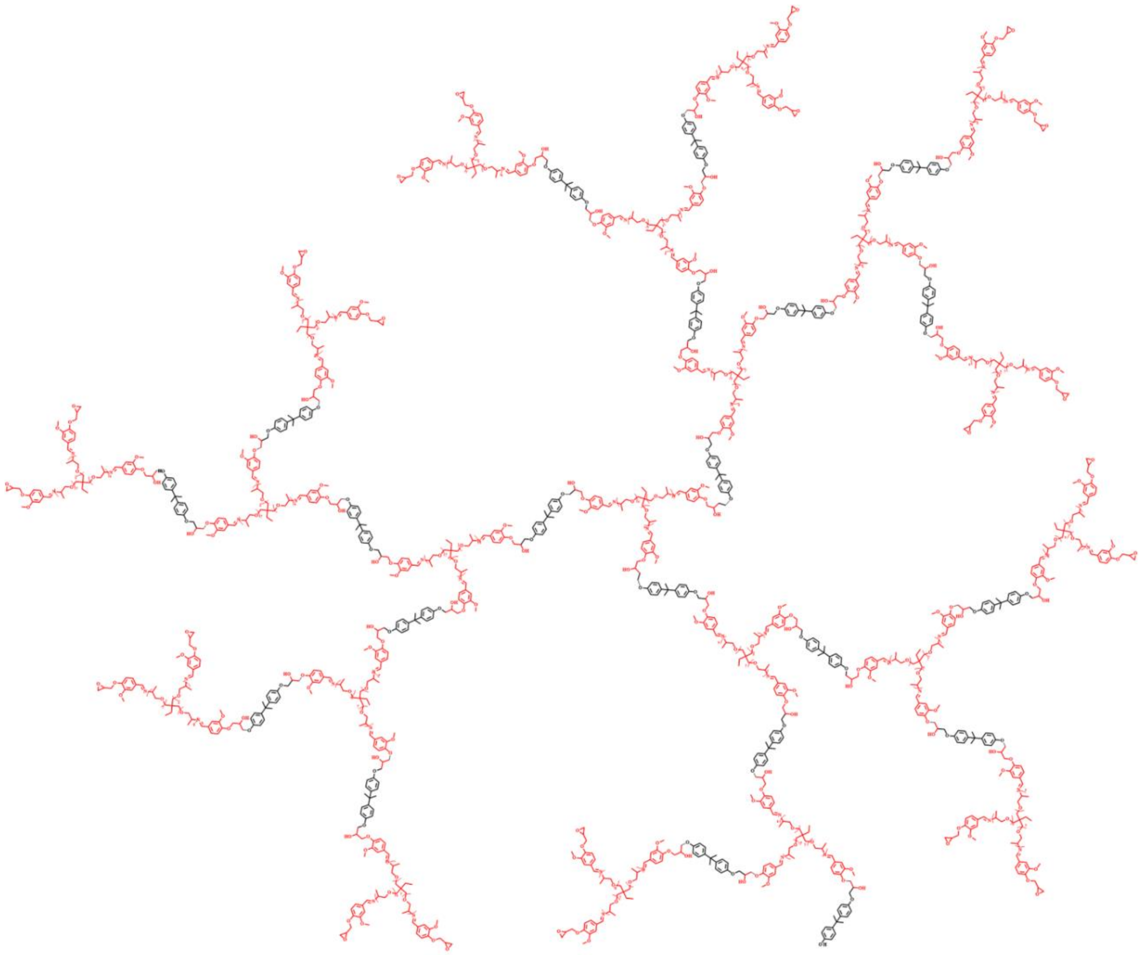

Supplementary Figure 2. Chemical structure of VanEHBP.

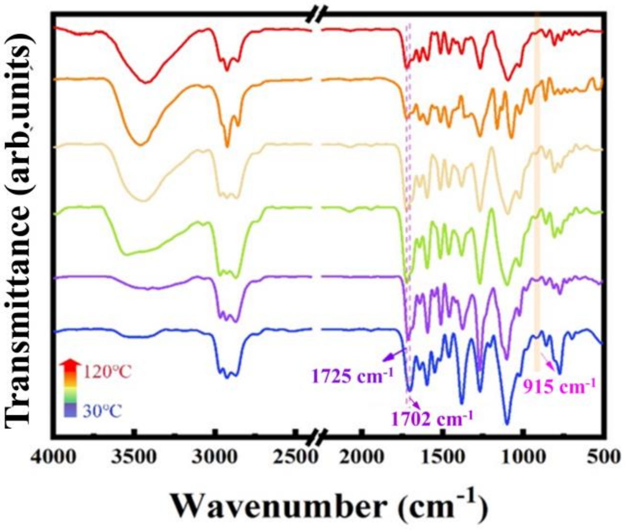

Supplementary Figure 3. Temperature-dependent FT-IR spectra of EN-VanEHBP7 upon heating from 30 to 120 °C (interval: 3 °C).

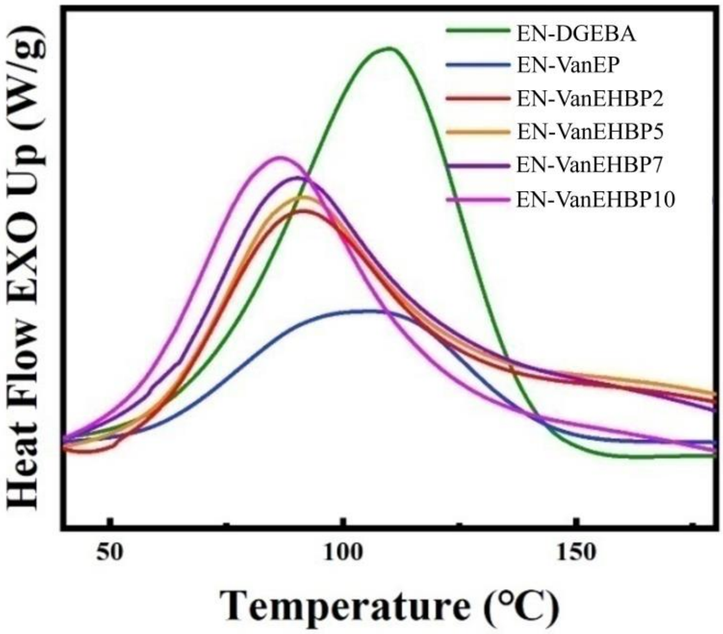

Supplementary Figure 4. DSC curves of the epoxy supramolecular thermosets from 40 to 180 °C with a heating rate of 10 °C·min^{-1}.

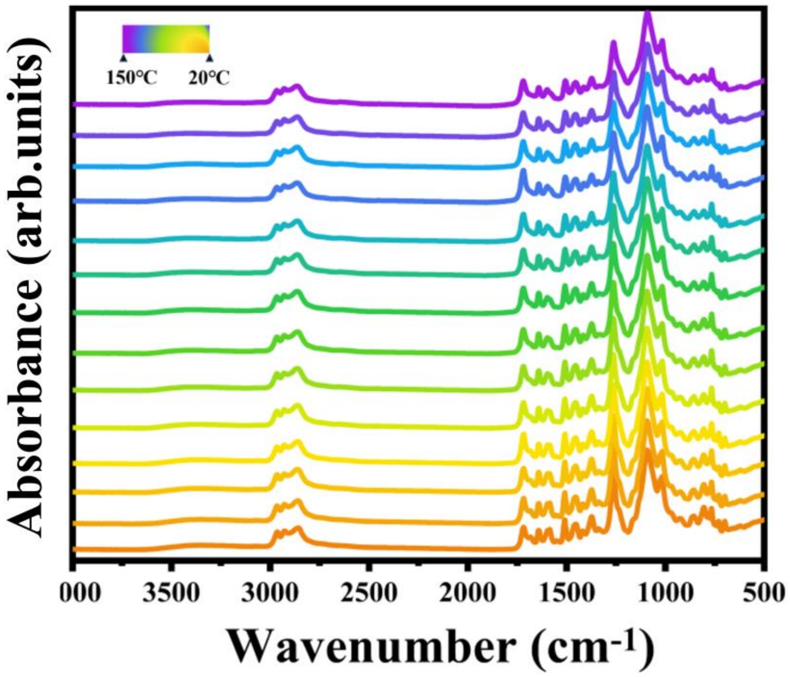

Supplementary Figure 5. Temperature-dependent FT-IR spectra of EN-VanEHBP7 upon heating from 20-150 °C.

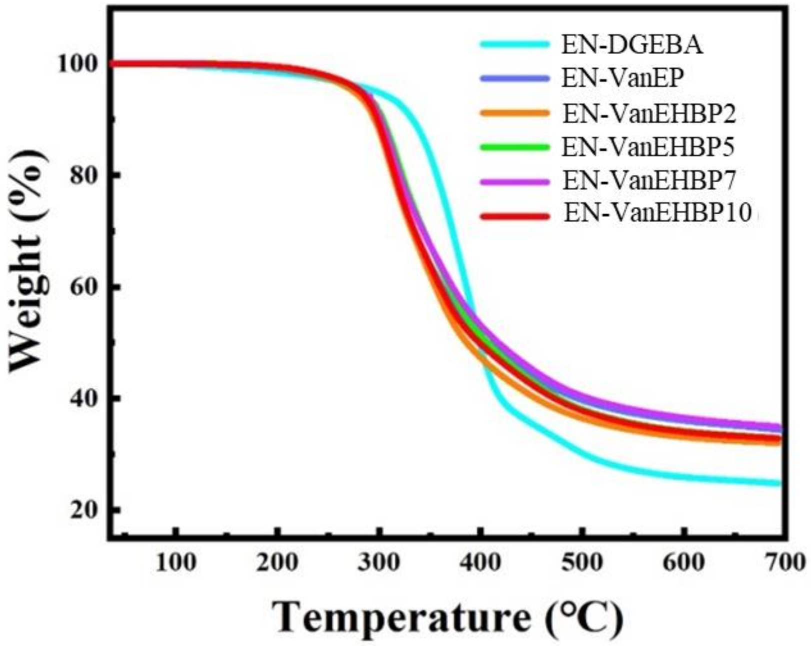

Supplementary Figure 6. TGA curves of the epoxy supramolecular thermosets from 30 to 700 °C with a heating rate of 10 °C·min^{-1}

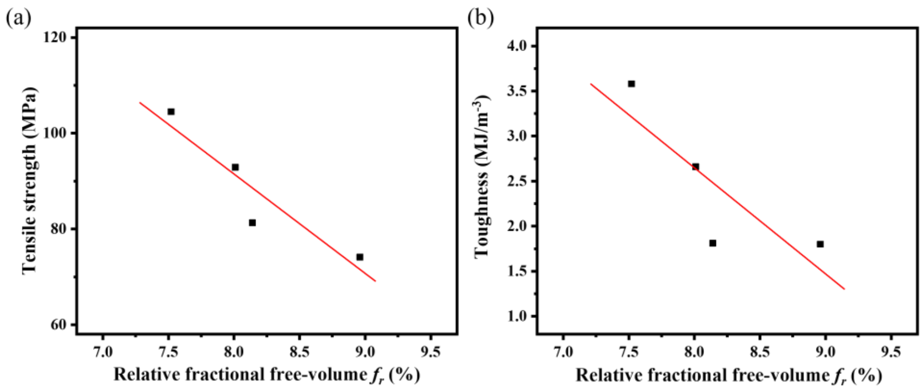

Supplementary Figure 7. Variations in (a) tensile strength and (b) toughness as functions of relative fractional free-volume f_r.

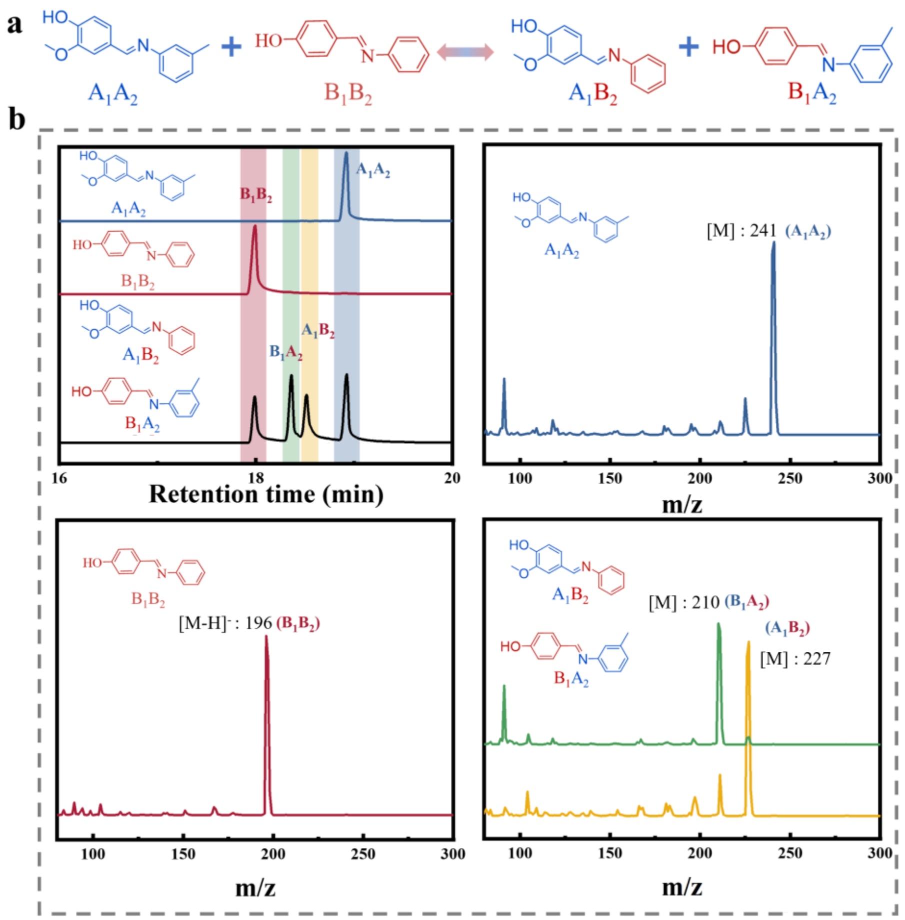

Supplementary Figure 8. (a) Exchange reaction of the model molecules. (b) GC-MS spectrum of model molecules and products of the imine exchange reaction at room temperature.

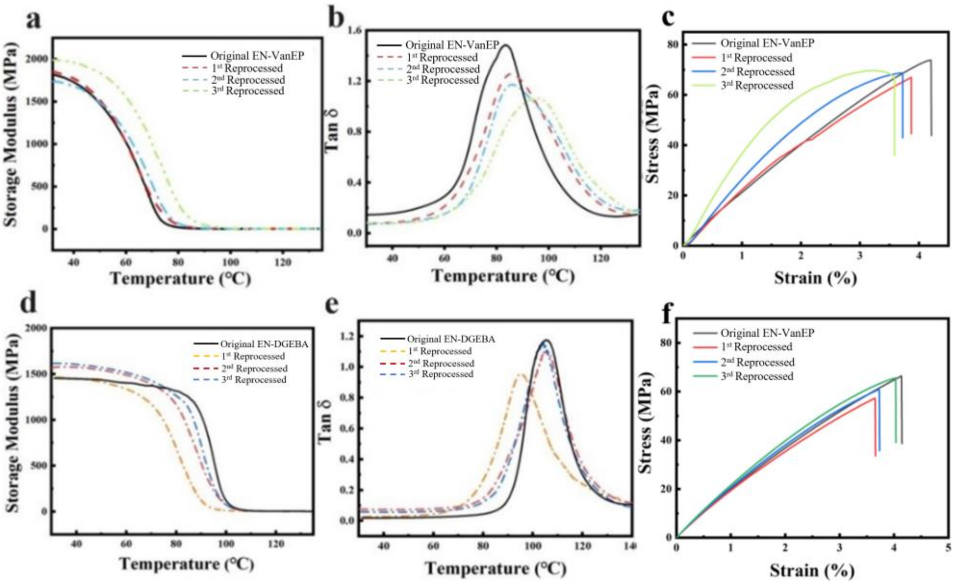

Supplementary Figure 9. Storage modulus (a), tan δ (b) and stress-strain curves (c) of the original and reprocessed EN-VanEP. Storage modulus (d), tan δ (e) and stress-strain curves (f) of the original and reprocessed EN-DGEBA. The error bars represent the standard deviations of the measured values, n = 3 independent samples. Source data are provided in the Source Data file.

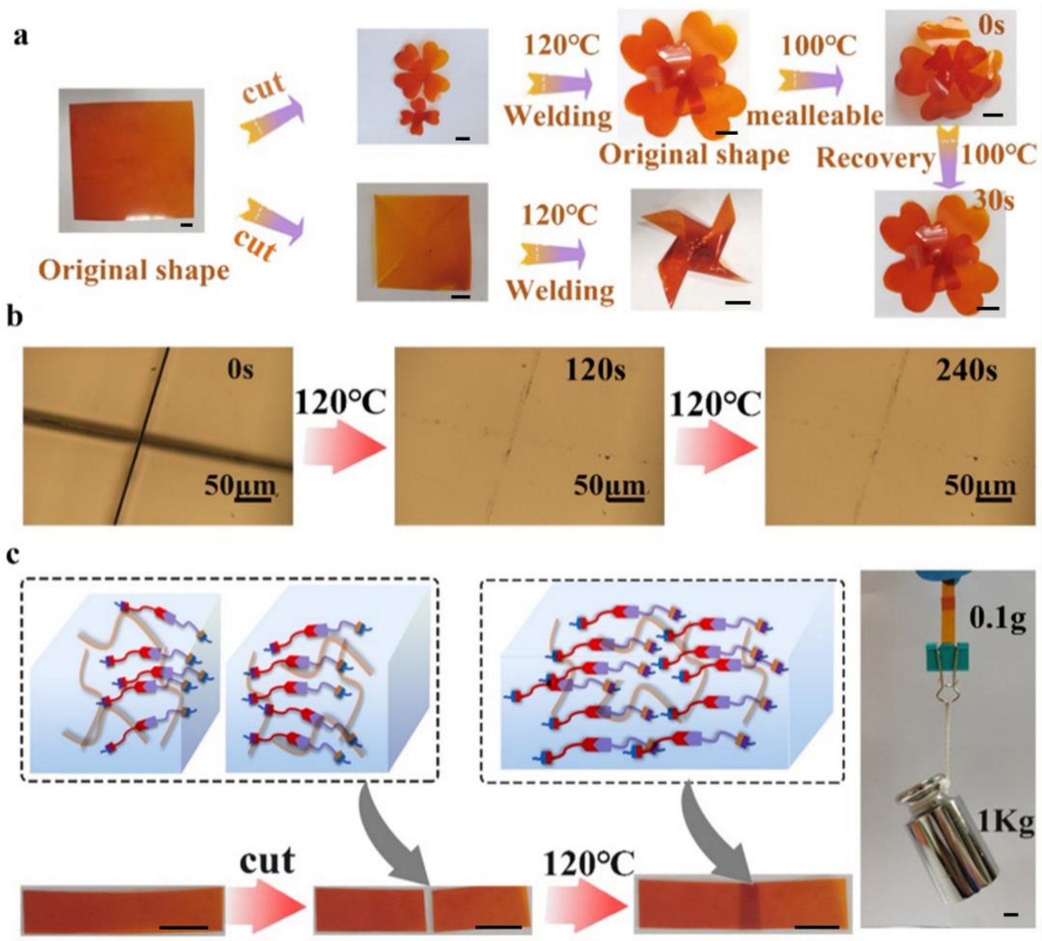

Supplementary Figure 10. (a) Photographs of EN-VanEHBP7 samples deformed at different temperatures, followed by shape recovery at different temperatures. Scale bar: 1 cm. (b) Optical microscopy images of the EN-VanEHBP7 scratch and self-healing experiments at 120 ℃. Scale bar: 1 cm. (c) Welding tests of EN-VanEHBP7 at 120 ℃. Scale bar: 1 cm.

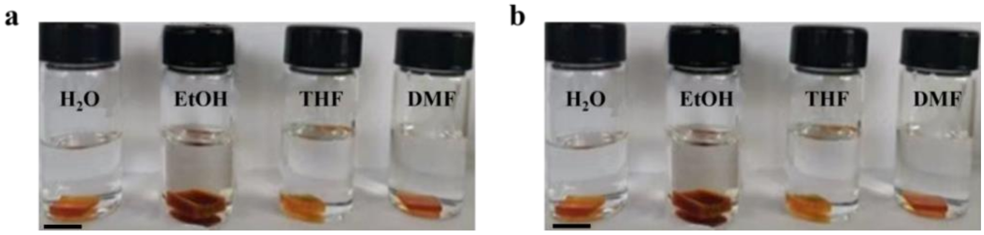

Supplementary Figure 11. Images of EN-VanEHBP7 in solvents at room temperature before (a) and after (b) 72 h. Scale bar: 1 cm.

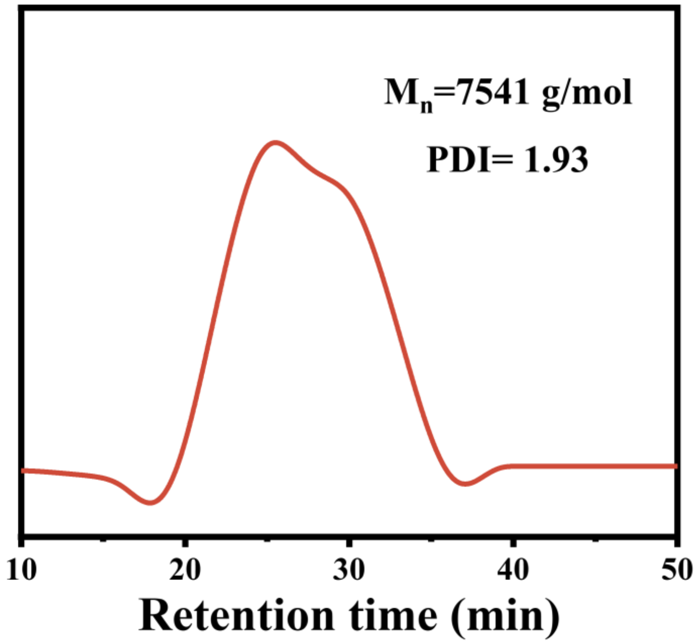

Supplementary Figure 12. GPC analysis of the degraded solution of EN-VanEHBP7**.** GPC was conducted in DMF using a RI signal with polystyrene (PS) standards.

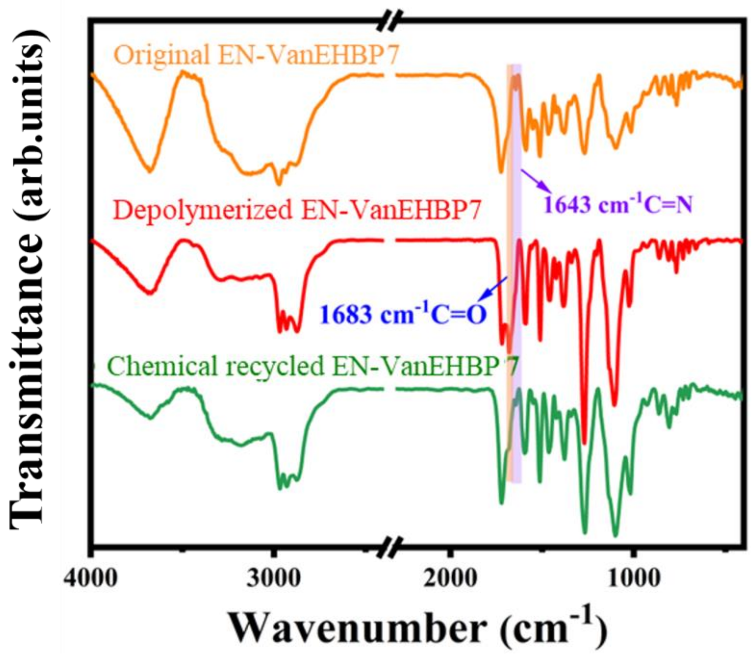

Supplementary Figure 13. FT-IR spectra of the original, depolymerized and chemically recycled EN-VanEHBP7.

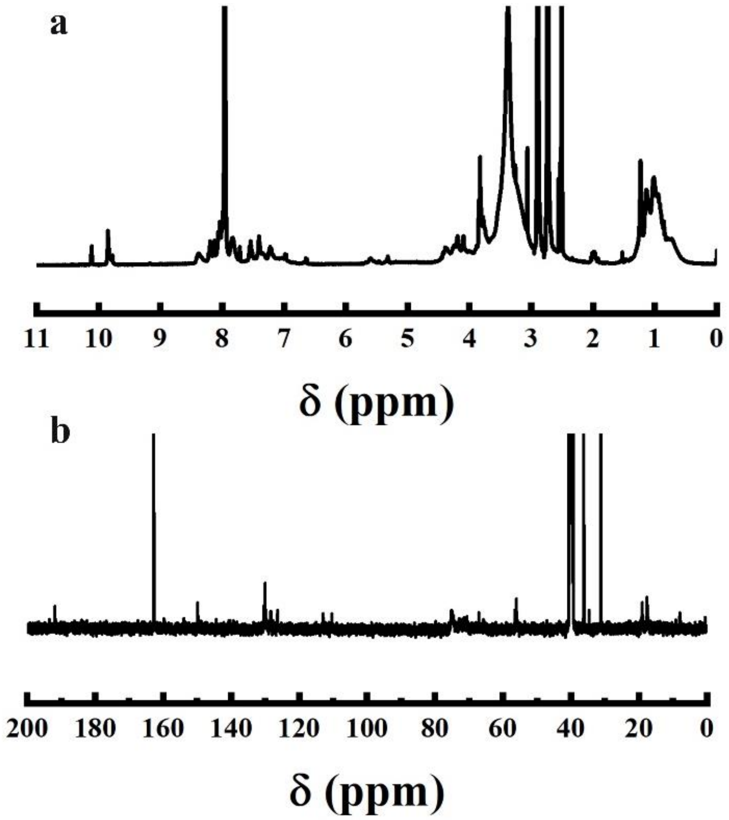

Supplementary Figure 14. (a) ^{1}H NMR (400 MHz) and (b) ^{13}C NMR (100 MHz) spectra of depolymerized EN-VanEHBP7 with 0.1 M HCl and DMF at room temperature in the mixture of DMSO-d_6, and the protein concentration was 40 μM.

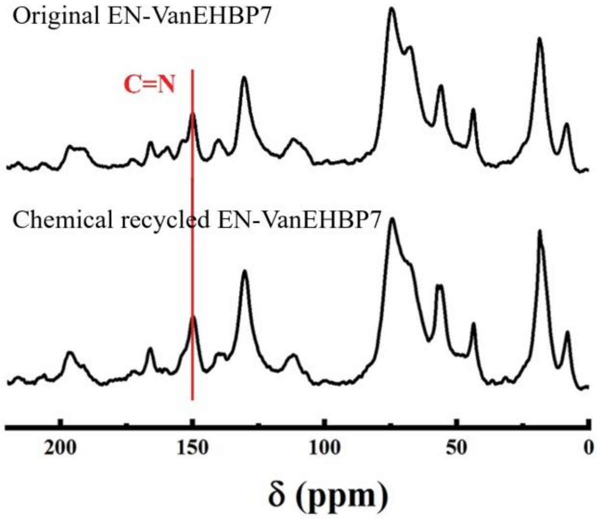

Supplementary Figure 15. Solid-state ^{13}C NMR spectra (100 MHz) of the original and chemically recycled EN-VanEHBP7.

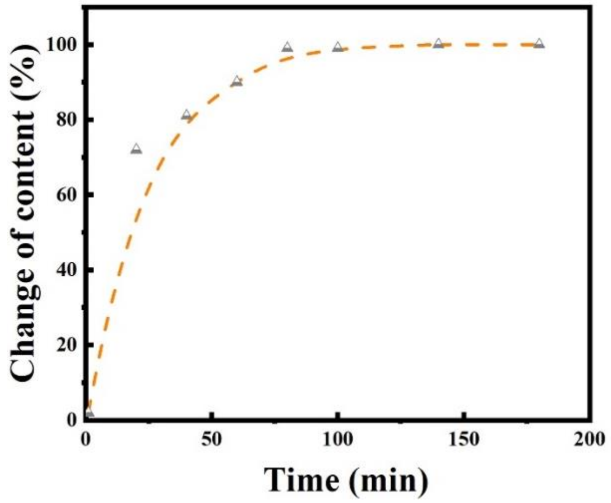

Supplementary Figure 16. Plot of the content of aldehyde groups as a function of time with 0.1 M HCl and DMF at room temperature in the mixture of DMSO-d_6.

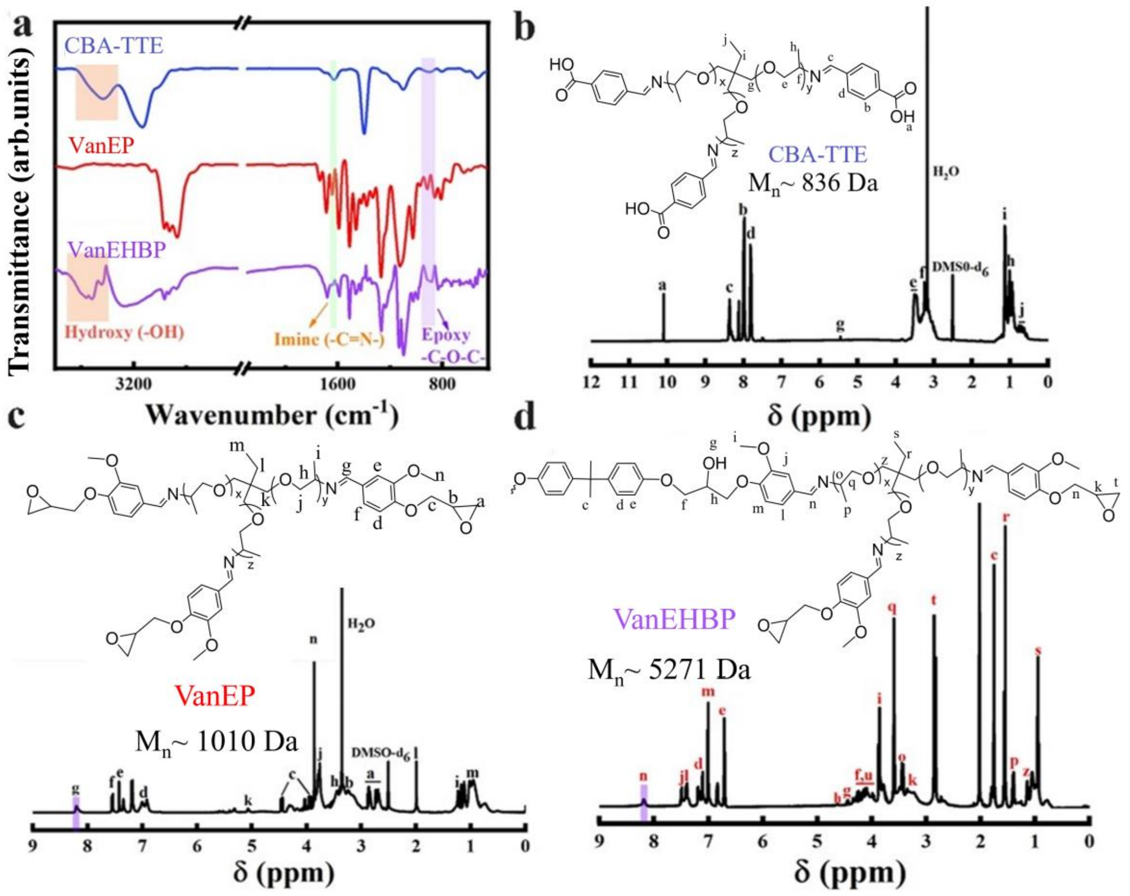

Supplementary Figure 17. (a) FT-IR spectra of CBA-TTE, VanEP and VanEHBP, ^{1}H NMR spectra of (b) CBA-TTE, (c) VanEP, and (d) VanEHBP. ^{1}H NMR measurements were performed at room temperature and 400 MHz using DMSO-d_6 as solvents, and the protein concentration was 40 μM.

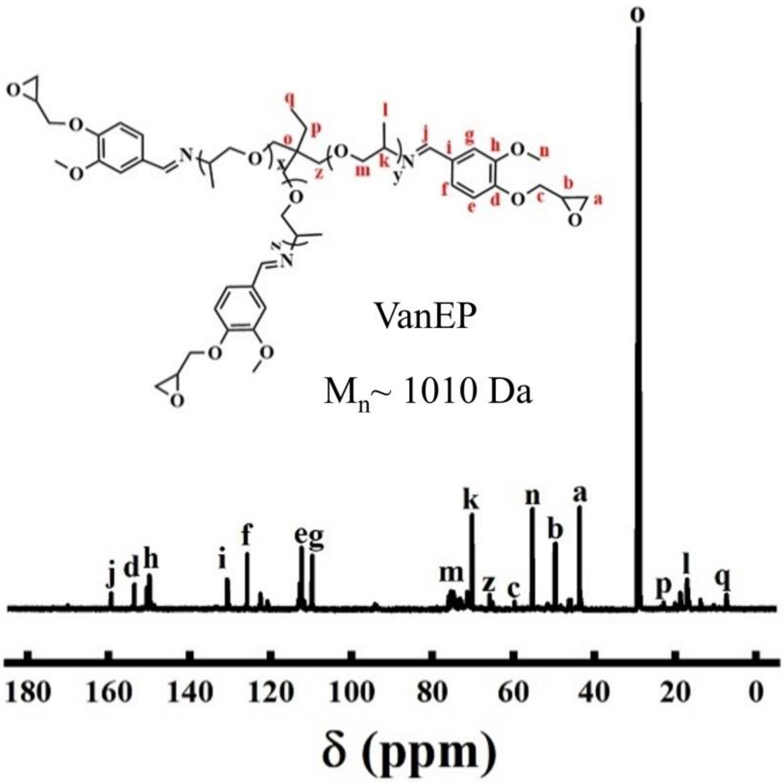

Supplementary Figure 18. ^{13}C NMR spectrum (100 MHz) of VanEP using $CDCl_3$ as solvents, and the protein concentration was 40 μM.

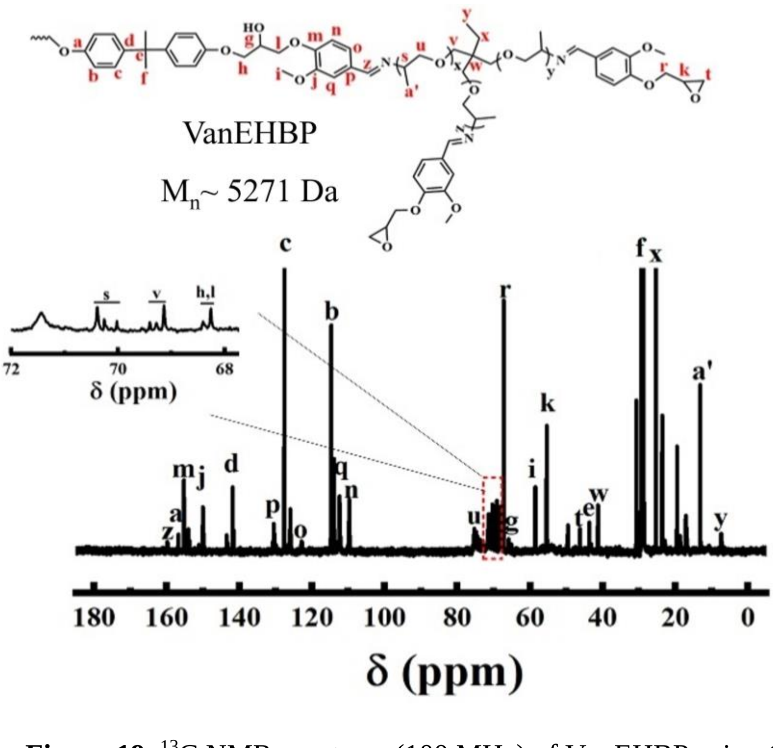

Supplementary Figure 19. ^{13}C NMR spectrum (100 MHz) of VanEHBP using $CDCl_3$ as solvents, and the protein concentration was 40 μM.

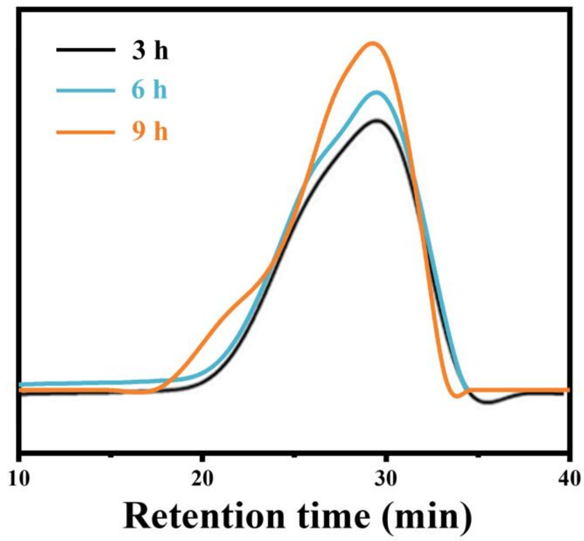

Supplementary Figure 20. Evolution of the molecular weight during the polymerization of VanEHBP. GPC was conducted in DMF using a RI signal with polystyrene (PS) standards.

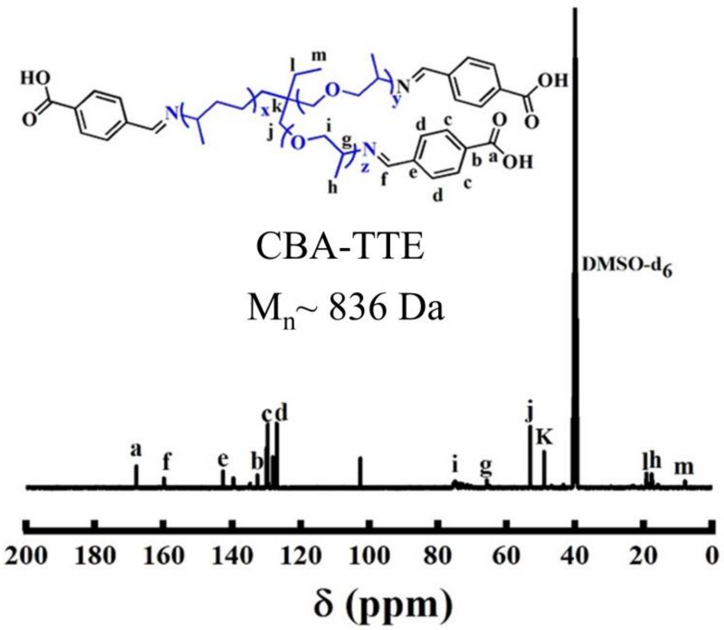

Supplementary Figure 21. ^{13}C NMR spectrum (100 MHz) of CBA-TTE using DMSO-d_6 as solvents, and the protein concentration was 40 μM.

Supplementary Table 1. DSC characteristic parameters of the epoxy supramolecular thermosets

Samples	T_i (°C)[a]	T_p (°C)[b]	ΔH (J•g^{-1})[c]
EN-DGENA	68	110	282
EN-VanEP	59	106	117
EN-VanEHBP2	59	91	140
EN-VanEHBP5	59	91	145
EN-VanEHBP7	58	89	197
EN-VanEHBP10	54	86	204

[a] T_i is the initial reaction temperature, [b] T_p is the peak reaction temperature, and [c] ΔH is the reaction activation energy.

Supplementary Table 2. Mechanical and thermomechanical properties of the epoxy supramolecular thermosets

Samples	Tensile Strength (MPa)	Toughness (MJ·m^{-3})	Young's Modulus (MPa)	Impact Strength (KJ·m^{-2})	E_c (GPa)	T_g (°C)	E_d (MPa)	ρ (×10^{-3}mol •cm^{-3})
EN-DGEBA	66.3±5.6	1.51±0.14	1832±334	24.0±2.7	1.5	105	2.9	0.29
EN-VanEP	73.0±4.8	1.71±0.09	1945±245	25.0±3.4	1.8	83	1.3	0.14
EN-VanEHBP2	74.1±3.2	1.80±0.12	1998±281	29.5±3.6	1.7	86	1.3	0.13
EN-VanEHBP5	92.9±3.1	2.66±0.10	2332±256	48.3±3.5	2.4	92	3.0	0.30
EN-VanEHBP7	104.5±3.4	3.58±0.08	2400±214	57.0±3.3	2.8	95	4.6	0.46
EN-VanEHBP10	81.3±3.2	1.81±0.13	2223±273	42.6±3.6	2.1	86	2.8	0.29

E_c is the storage modulus at 30 °C, T_g is the glass transition temperature, E_d is the storage modulus at T_g + 30 °C, and ρ is the crosslinking density.

Supplementary Table 3. TGA data of the epoxy supramolecular thermosets under a nitrogen atmosphere

Samples	$T_{d5\%}$ (°C)	$T_{d10\%}$ (°C)	T_{dmax} (°C)	$Char_{700}$ (%)
EN-DGEBA	298	333	392	25
EN-VanEP	281	298	317	33
EN-VanEHBP2	280	296	313	33
EN-VanEHBP5	280	297	315	33
EN- VanEHBP7	283	302	314	35
EN- VanEHBP10	281	298	313	33

$T_{d5\%}$ and $T_{d10\%}$ are the 5 % and 10 % weight loss temperature, respectively, T_{dmax} is the temperature at which the degradation rate reached a maximum, and $Char_{700}$ is the char yield at 700 °C.

Supplementary Table 4. PAL data and free volume fraction of the epoxy supramolecular thermosets

Samples	I_3 (%)	R (Å)	f_r (%)
EN-DGEBA	8.74	2.72	13.35
EN-VanEP	5.68	2.55	7.07
EN-VanEHBP2	5.81	2.73	8.89
EN-VanEHBP5	6.33	2.56	7.97
EN-VanEHBP7	6.05	2.55	7.52
EN-VanEHBP10	5.32	2.73	8.13

Supplementary Table 5. The stress relaxation experimental data of the epoxy supramolecular thermosets

Samples	Relaxation time (s)				E_a (kJ•mol^{-1})	Average value E_a (kJ•mol^{-1})
	130 °C	120 °C	110 °C	100 °C		
EN-DGEBA	18.0	22.0	31.5	42.2	44.8	47.5 ± 2.8
	16.1	21.3	30	43.6	50.3	
	17.8	22.1	33.5	44.1	47.2	
	17.4	21.7	32.0	43.3	47.7	
VanEP	14.1	21.4	26.3	40.6	40.6	45.5 ± 4.9
	15.2	20.3	27.5	40.1	45.1	
	14.5	20.5	26.1	39.0	48.3	
	15.0	20.1	27.2	38.0	48.1	
EN-VanEHBP7	12.0	14.5	17.0	20.3	26.1	27.4 ± 2.7
	12.4	15.1	18.1	22.1	28.6	
	11.6	15.0	17.9	21.5	30.1	
	12.3	14.9	18.0	22.1	24.9	

Supplementary Table 6. Tensile strength of original, reprocessed and chemical recycled epoxy supramolecular thermosets

Samples		EN-DGEBA	EN-VanEP	EN-VanEHBP7
Original	Tensile strength (MPa)	66.3 ± 5.6	73.0 ± 4.8	104.5 ± 3.4
1stReprocessing cycled	Tensile strength (MPa)	57.2 ± 2.0	66.9 ± 1.4	104.0 ± 2.8
	Recovery efficiency (%)	86.3	94.2	99.5
2ndReprocessing cycled	Tensile strength (MPa)	60.7 ± 3.8	68.6 ± 1.1	104.0 ± 4.7
	Recovery efficiency (%)	91.5	94.6	99.5
3rdReprocessing cycled	Tensile strength (MPa)	65.5 ± 4.9	69.1 ± 1.5	104.3 ± 1.0
	Recovery efficiency (%)	98.7	95.2	99.8
Chemical recycled	Tensile strength (MPa)	54.2 ± 1.3	65.9 ± 3.1	105.5 ± 2.1
	Recovery efficiency (%)	81.7	90.3	100.5

Supplementary Table 7. DMA data of the original, reprocessed and chemically recycled EN-DGEBA

Samples	E_c (GPa)	T_g (°C)	E_d (MPa)	ρ ($\times 10^{-3}$ mol·cm^{-3})
Original	1.5	105	2.9	0.29
1stReprocessing cycled	1.5	95	2.8	0.28
2ndReprocessing cycled	1.6	106	2.5	0.25
3rdReprocessing cycled	1.6	104	3.2	0.31
Chemically recycled	1.4	99	2.0	0.20

Supplementary Table 8. DMA data of the original, reprocessed and chemically recycled EN-VanEP

Samples	E_c (GPa)	T_g (°C)	E_d (MPa)	ρ ($\times 10^{-3}$ mol·cm^{-3})
Original	1.8	83	1.3	0.14
1stReprocessed	1.8	85	1.0	0.10
2ndReprocessed	1.7	85	1.0	0.10
3rdReprocessed	1.9	94	1.3	0.13
Chemical recycled	1.6	82	1.0	0.10

Supplementary Table 9. DMA data of the original, reprocessed and chemically recycled EN-VanEHBP7

Samples	E_c (GPa)	T_g (°C)	E_d (MPa)	ρ ($\times 10^{-3}$ mol·cm^{-3})
Original	2.8	95	4.6	0.46
1st Reprocessed	2.7	94	3.2	0.32
2nd Reprocessed	2.6	90	4.2	0.43
3rd Reprocessed	2.6	98	4.4	0.44
Chemical recycled	2.8	93	4.5	0.45

Supplementary Table 10. The swelling ratio and gel fraction of the epoxy supramolecular thermosets

Samples	Swelling ratio(%)						Gel fraction (%)
	H_2O	EtOH	THF (25°C)	DMF (25°C)	THF (65°C)	DMF (120°C)	
EN-DGEBA	1.6	1.5	1.4	1.9	14.7	35.3	95.0
EN-VanEP	2.5	5.0	1.5	2.1	13.6	44.0	94.1
EN-VanEHBP7	1.3	4.5	0.7	1.2	12.0	30.4	95.6

Supplementary Table 11. Composition of the epoxy supramolecular thermosets

Samples	DGEBA (g)	VanEP (g)	VanEHBP (g)	CBA-TTE (g)
EN-DGEBA	10.00	0.00	0.00	15.81
EN-VanEP	0.00	10.00	0.00	8.37
EN-VanEHBP2	0.00	10.00	0.20	8.48
EN-VanEHBP5	0.00	10.00	0.50	8.65
EN-VanEHBP7	0.00	10.00	0.70	8.76
EN-VanEHBP10	0.00	10.00	1.00	8.93

Supplementary Table 12. GPC data for molecular weight determination and epoxy value of VanEHBP

Polymerization time (h)	M_n (g•mol^{-1})	M_w (g•mol^{-1})	PDI	Epoxy value (mol 100•g^{-1})
3	3519	5806	1.65	0.21
6	5271	9172	1.74	0.18
9	5405	11567	2.14	0.12
